# Supplementary material for: Identifying Predictors of Changes in Physical Activity Level in Adolescence: A Prospective Analysis in Bosnia and Herzegovina
Source: Int J Environ Res Public Health. 2019 Jul 18;16(14):2573. doi: 10.3390/ijerph16142573 (PMC6679343; doi:10.3390/ijerph16142573)
Supplement: Supplementary file 1 [file ijerph-16-02573-s001.pdf]

Supplementary table 1. Frequencies for studied variables

	Total		Females		Males	
	F	%	F	%	F	%
Urban/Rural						
Rural	548	62.8%	252	62.4%	288	62.6%
Urban	324	37.2%	152	37.6%	172	37.4%
Socioeconomic status						
Below average	14	1.6%	2	0.5%	12	2.6%
Average	812	93.1%	390	96.5%	414	90.0%
Above average	46	5.3%	12	3.0%	34	7.4%
Paternal education						
Elementary school	64	7.3%	42	10.4%	22	4.8%
High school	636	72.9%	292	72.3%	336	73.0%
College level	96	11.0%	40	9.9%	56	12.2%
University level	76	8.7%	30	7.4%	46	10.0%
Maternal education						
Elementary school	266	30.5%	150	37.1%	116	25.2%
High school	495	56.8%	212	52.5%	283	61.5%
College level	50	5.7%	22	5.4%	28	6.1%
University level	61	7.0%	26	6.4%	35	7.6%
Smoking						
Never smoked	580	66.5%	280	69.3%	294	63.9%
Quitted	44	5.0%	14	3.5%	28	6.1%
From time to time but not daily	160	18.3%	74	18.3%	86	18.7%
Daily smoking (< 10 cigs)	42	4.8%	20	5.0%	22	4.8%
Daily smoking (> 10 cigs)	46	5.3%	16	4.0%	30	6.5%
AUDIT CAT						
Nonharmful drinking	772	88.5%	374	92.6%	390	84.8%
Harmful drinking	100	11.5%	30	7.4%	70	15.2%
Illicit drugs						
Nonusers	830	95.2%	390	96.5%	440	95.7%
Users	34	3.9%	14	3.5%	20	4.3%
PA_changes						
incline	336	38.5%	146	36.1%	182	39.6%
decline	536	61.5%	258	63.9%	278	60.4%
